# The rhythm of memory. Does theta frequency audio/visual flicker improve recall?

**DOI:** 10.3389/fnbeh.2025.1555081

**Published:** 2025-03-13

**Authors:** Louise Simeonov, Ravi Das

**Affiliations:** Clinical Psychopharmacology Unit, Research Department of Clinical, Educational, and Health Psychology, University College London, London, United Kingdom

**Keywords:** memory, oscillations, neural entrainment, theta, association, LTP, LTD

## Abstract

A fundamental question in cognitive neuroscience is how multi-sensory elements are bound into a unified memory trace. The formation of memories is thought to be reliant on precisely timed neural activity. Theta frequency neural oscillations have been proposed to orchestrate the timing of different sensory cortices. Here, we attempt to replicate findings that flickering stimuli presented in synchronous theta frequency will lead to enhanced recall. Additionally, we investigate whether theta frequency sensory flicker can improve encoding of emotional associative memories. The current study failed to replicate previous findings demonstrating improved recall for stimuli synchronously modulated at theta frequency in a multi-sensory associative learning task. We discuss possible explanations for the discrepancy between current and previous findings.

## 1 Introduction

The collection of sensory elements that constitute an episodic memory require processing across a distributed set of regions across the cortex as they are formed and recalled. These elements are bound into a unified memory trace or “engram,” such that on recalling a memory we not only remember sights but also sound, smells, tastes and more (Shing et al., 2010; Treisman, 1996; Zimmer et al., 2006). A fundamental question in cognitive neuroscience is *how* these multi-sensory elements are unified, and whether this binding can be enhanced or diminished to impact encoding and recall of memory. Non-invasive means of doing so could be useful mnemonic aids or potentially used to suppress unwanted memory associations.

At the cellular level, the formation of new memories is thought to require tuning of synaptic connectivity via long term potentiation or depression (LTP/LTD). Connective weights between synapses are then thought to be stabilised via a protein-synthesis-dependent process of consolidation, which produces enduring memory traces that can be accessed at remote time points. Brain oscillations: the rhythmic fluctuation in the excitability of neuronal populations; are thought to be key to the synchronised neural firing that is required for coordinating spike-timing dependent plasticity. The role of the synchronisation/desynchronisation of neuronal populations (Hanslmayr et al., 2016) in memory is complex, however the hippocampus is centrally implicated in coordinating these oscillatory “binding” processes. The hippocampus receives inputs from most sensory cortices and acts as a neural “switchboard” for controlling the connectivity of these regions. It has been proposed that hippocampal theta oscillations (4–8 Hz) serve to organise multiple neural inputs, and therefore facilitate enhancements in synaptic strength (Muzzio et al., 2009) and the formation of memories (Buzsáki, 2005). Evidence from rodent studies has shown that stimulation of the hippocampus can lead to LTP or LTD depending on *where* in theta phase the stimulation is applied (Hölscher et al., 1997; Huerta and Lisman, 1995; Pavlides et al., 1988). Given the evolutionary conservation of neural oscillations, we may expect theta rhythms to impact human memory, however, direct evidence for a theta-dependent memory effect in humans is more scarce, partly due to the invasiveness of procedures used to induce and measure oscillatory activity in rodents.

A non-invasive means to investigate the causal impact of brain oscillations on memory is via the sensory entrainment effect (Herrmann, 2001; Thut et al., 2011). Neuronal assemblies in sensory regions are known to synchronise to the frequencies and phase of external sensory “entrainment” stimuli, such as a 4 Hz auditory tone, or flickering pattern (Becher et al., 2015). Clouter et al. (2017) used two stimulus elements (auditory and visual), modulated such that they were either synchronous (phase offset of 0°) or asynchronous (phase offset of 180°), in an associative learning paradigm. When auditory and visual stimuli were in a synchronous theta (4 Hz) phase, greater recall of the association between auditory and visual elements was observed. These findings were replicated by the same research group (Wang et al., 2018), providing the first causal evidence of a theta entrainment effect on memory performance in humans.

The ability to enhance associative binding with simple, non-invasive procedures could be of great utility in improving learning and may offer insights into deficits in associative learning associated with certain neurological conditions. However, it is unclear what the practical limits of this effect may be. For example, it may be observable in highly noise-controlled lab environments over many trials, but not in naturalistic environments or at the single-item level. Properties of to-be-learned stimuli may also play a role. Clouter et al. (2017) used affectively neutral stimuli. However, emotionality effects on encoding of material are well documented, with emotive material more strongly encoded (Kensinger, 2009) and more easily recalled. Furthermore, it is of interest to assess the ecological robustness of theta-induced memory effects (TIME) outside of the highly controlled lab environment, as these are the contexts in which naturalistic learners are likely to encounter to-be-learned material.

As such, we aimed to replicate and extend the findings of Clouter et al. (2017) and Wang et al. (2018) by assessing whether the TIME is abolished by the by the use of emotive/neutral images; something we have previously demonstrated for retrieval-suppression effects with salient reward stimuli (Simeonov et al., 2022).

This study was originally intended to be performed in-person, with collection of EEG data to test the success of oscillatory entrainment. However, due to the COVID-19 pandemic, all in-person testing activities were suspended and as such the data collection had to be performed online, via the online psychological testing platform Pavlovia. While this was not ideal in terms of stimulus control and neural data validation, it allowed us to test the ecological robustness of the effect in variable environments (participants’ own homes), where “naturalistic” learning (e.g., studying for an exam) would typically take place.

## 2 Methods

### 2.1 Participants

Participants were 42 adults aged between 18–35. A power analysis was conducted using an effect size of *d* = 1.3 from the flicker vs. no-flicker control experiment from Clouter et al. (2017), as this is the most similar analysis to the current study. To achieve 95% power at *α* = 0.05 in a 2 by 3 within subject design a sample of six participants would be needed. Exclusion criteria were: a current diagnosis of any psychiatric disorder, a score of >14 on the Insomnia Severity Index (Morin, 1993) and any condition that may be triggered by flashing lights (e.g., photosensitive epilepsy). Inclusion criteria were: English language fluency, normal hearing and colour vision. Participants were fully informed of the use of emotive images prior to taking part in the study. Eligibility was assessed using an online screening survey and telephone screen. All participants gave informed consent and all procedures for the study were approved by the UCL Research Ethics Committee (The ethics ID is 21091/001). All participants were paid for their participation in the study.

### 2.2 Design and procedure

This was a 2 (emotive/neutral images) × 3 (theta phase, alpha phase, no modulation) within-subjects design. Eligible participants were contacted by telephone to verbally explain the study and verify intention to participate. The learning and test procedures were then completed online using Pavlovia (Peirce et al., 2019). The study lasted approximately 1.5 h and was always completed between 9 am and 7 pm. The participants first completed questionnaires and then continued to the task.

### 2.3 Questionnaire measures

The Positive and Negative Affect Scale Short Form (PANAS-SF) (Thompson, 2007) was used to assess participants’ mood and the State-Trait Anxiety Inventory (STAI; Spielberger et al., 1970) to assess trait anxiety. The Insomnia Severity Index (Morin, 1993) includes seven questions rated on a Likert scale. Scores were used to assess that no participant suffered from clinical insomnia.

### 2.4 Stimuli

#### 2.4.1 Images

Forty eight negative emotive images were sourced from International Affect Picture System (Lang and Bradley, 2007) (IAPS; arousal >4, valence <2.5). The neutral images were a single frame taken from the video stimuli set used by Clouter et al. (2017). A single frame was taken to match the stimuli more closely to the emotive images.

Following Clouter et al. (2017), the images appeared on screen for either 1.5 s (unmodulated) or 3 s (modulated). This was to equate the total visible/audible stimulus latencies across the two conditions; as the flickering stimuli alternated between visible/invisible and audible/inaudible. The modulated images were luminance-modulated using a sine wave at either 4 Hz (theta) or 10 Hz (alpha), such that the images appeared to “flicker” from no luminance to full luminance in the relevant frequency. Thirty-two (16 emotive and 16 neutral) images were modulated using a 4 Hz sine wave, 32 (16 emotive and 16 neutral) were modulated using a 10 Hz sine wave and 32 were left unmodulated (16 emotive and 16 neutral).

#### 2.4.2 Sounds

The auditory stimuli were the same as those used in Clouter et al. (2017) and were kindly provided by the authors. These clips were taken from Apple Loops for Garage Band (6.0.5) and iLife Sound Effects audio libraries and from various unique soundtracks and were chosen such that amplitude modulation and rhythmic beats within the sounds were minimal. There were 96 different sounds which are from four different instrument categories: guitar, synth, acoustic and orchestra. As with image stimuli, 32 of the sounds were amplitude modulated using a 4 Hz sine wave, 32 were modulated using a 10 Hz sine wave and 32 were left unmodulated.

### 2.5 Associative memory task

**Figure 1:**
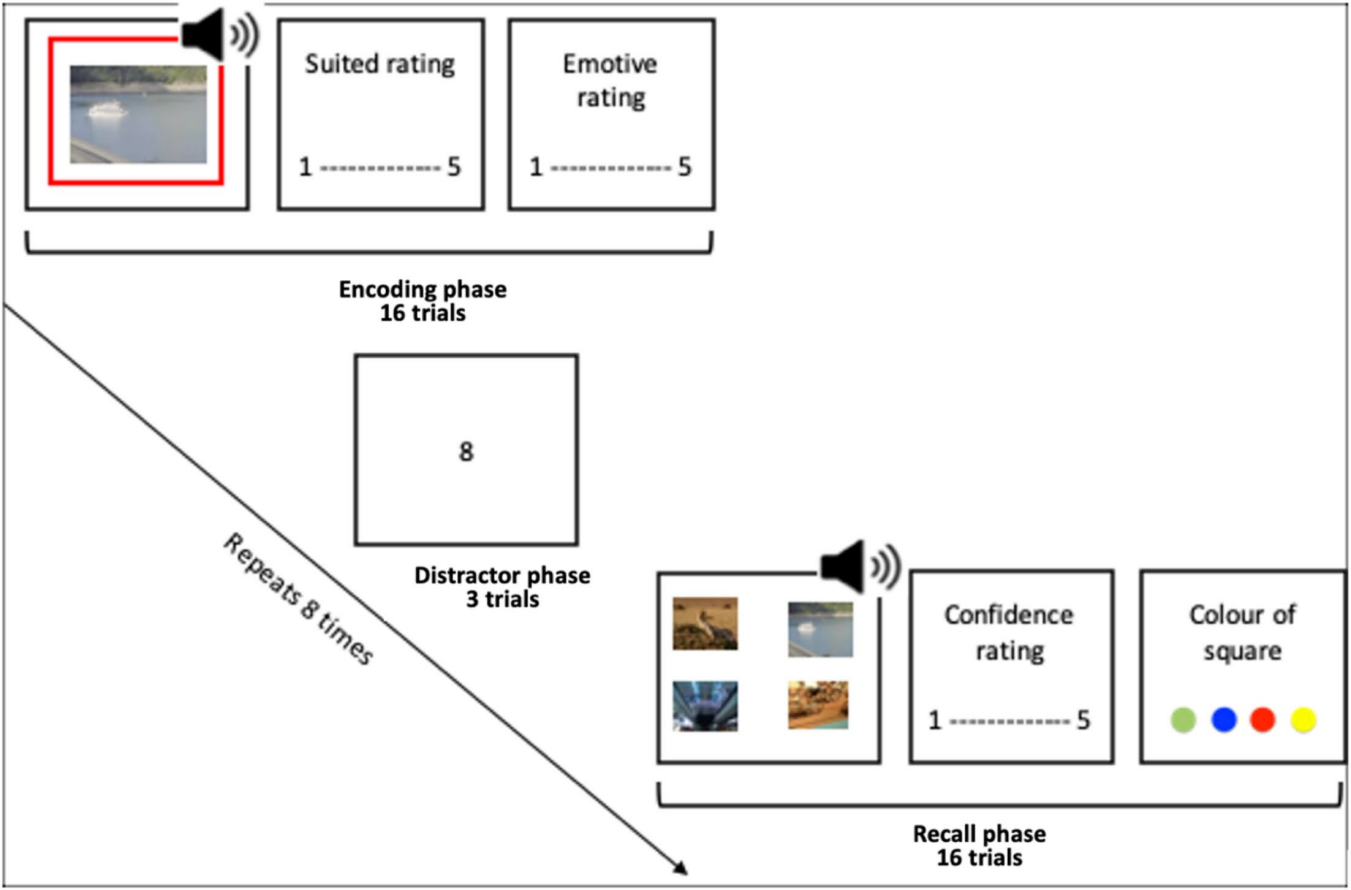
Encoding phase, a distractor task and a memory test.

The associative memory task consisted of six blocks. Each block consists of an encoding phase, a distractor task and a memory test (see Figure 1).

#### 2.5.1 Encoding task

Participants were instructed that their task was to try to remember some image-audio pairs, and that their recall of the pairs would be tested. They were also informed that they would be asked to rate how well the image suited the audio in each pair. This was to encourage participants to pay attention to the image and the audio. Participants were shown the image which was either (1) displayed unmodulated for 1.5 s whilst the audio played unmodulated, (2) had both the audio amplitude and image luminance modulated with a 4 Hz sine wave or (3) with a 10 Hz sine wave (in phase). The modulated stimuli were presented with zero-phase lag between the audio level and the luminance level. Such that when the luminance of the image was brightest the audio volume was loudest, and when the audio was the most quiet the luminance was at its lowest. An offset between images and sounds was introduced to account for the typical difference in processing speed between sound and vision, such that the visual led the onset of the sound clips by approximately 40 ms—as in Clouter et al. (2017). This aimed to lead to theta synchronisation in neural times eries, rather than absolute/native presentation time series. The frame rate was recorded as >57 for all except four participants. There are known issues with the java-script method used by Pavlovia to record frame rate and as such these participants were not immediately excluded. Instead, the analysis was conducted with and without these participants which showed no notable differences in the results.

A green, blue, yellow or red square outlined the image on each trial, to be used in an incidental memory test. On each trial, participants rated how well the audio and image were suited on a scale from “not suited at all” to “very well suited.” As well as rating the emotive content of the image on a scale with anchors “negative,” “neutral” and “positive.” In each block, this sequence repeated for 16 trials.

The stimuli were split into blocks of 16 trials, with two theta (4 Hz) modulated blocks, two 10 Hz modulated blocks and two unmodulated blocks. The order of blocks and the order of the trials within the blocks were randomised for each participant. The emotive and non-emotive stimuli were evenly distributed throughout the blocks. Additionally, the border square colour and audio instrument categories were counterbalanced throughout the blocks and the emotive/modulation conditions.

In an attempt to ensure the timing of stimulus presentation was accurate, tests were conducted using various web browsers and using different stimulus presentation packages. Pavlovia (Psychopy) in combination with Firefox (Mozilla, US) produced the best combination for timing accuracy in the researcher’s tests, in line with Bridges et al. (2020). Therefore, participants were asked to use the web browser Firefox and compliance with this was checked. Participants were asked to adjust the volume level such that it was “*Fairly loud, but not so loud it causes discomfort*.”

#### 2.5.2 Distractor task

Between each block, the participants completed the distractor task to prevent rehearsal. This was an adapted version of the digit span task. In this task single digits appeared sequentially in the centre of the screen. After 4, 5 or 6 digits appear the participant was asked to type out the digits. This repeated 3 times such that each participant received a number series of each length. This task took approximately 1 min.

#### 2.5.3 Recall task

During the recall task the audio was played, and participants were shown four images to choose the associated image from. The four images to choose from had been paired with the same music type (acoustic, synth, guitar or orchestral). There were always two emotive images and two neutral images to choose from. All the images shown had appeared in the encoding block just completed. Every image was shown an equal number of times. Participants were also asked how confident they were in their answer and to select what colour square they believe bordered the image.

Between each block participants were allowed to take a short break but were informed that no break should be greater than 5 min.

## 3 Data and analysis

The effects of stimuli flicker modulation and emotive content on memory performance were assessed using a repeated-measures ANOVA. Assumptions of normal distribution and sphericity were met. There were no extreme outliers. Data was aggregated using MATLAB and analysed using IBM SPSS.

## 4 Results

Participant demographic information is given in Table 1.

**Table 1:** Demographic information.

Measure	Mean (SD)/frequency
Age	24.26 (3.78)
Gender	*F* = 21; M = 21
Insomnia	4.17 (3.79)
STAI trait	38.76 (11.45)
PANAS—positive	26.57 (7.55)
PANAS—negative	14.17 (6.20)

STAI, State Trait Anxiety Index; PANAS, Positive and Negative Affect Scale Short Form.

Participants’ overall recall rate varied from 23 to 64 correct out of 96 trials (23.96–66.67%). The mean overall recall rate was 39.24 (40.87%), SD = 8.78 (chance level = 25%). A one-sample *t*-test confirmed that performance was significantly above chance level [*t*(41) = 10.51, *p* < 0.001]. The content rating confirmed that participants did find the emotive content (Mean = 1.66, SD = 0.32) more aversive than the neutral content (Mean = 3.19, SD = 0.23).

### 4.1 Recall

**Figure 2:**
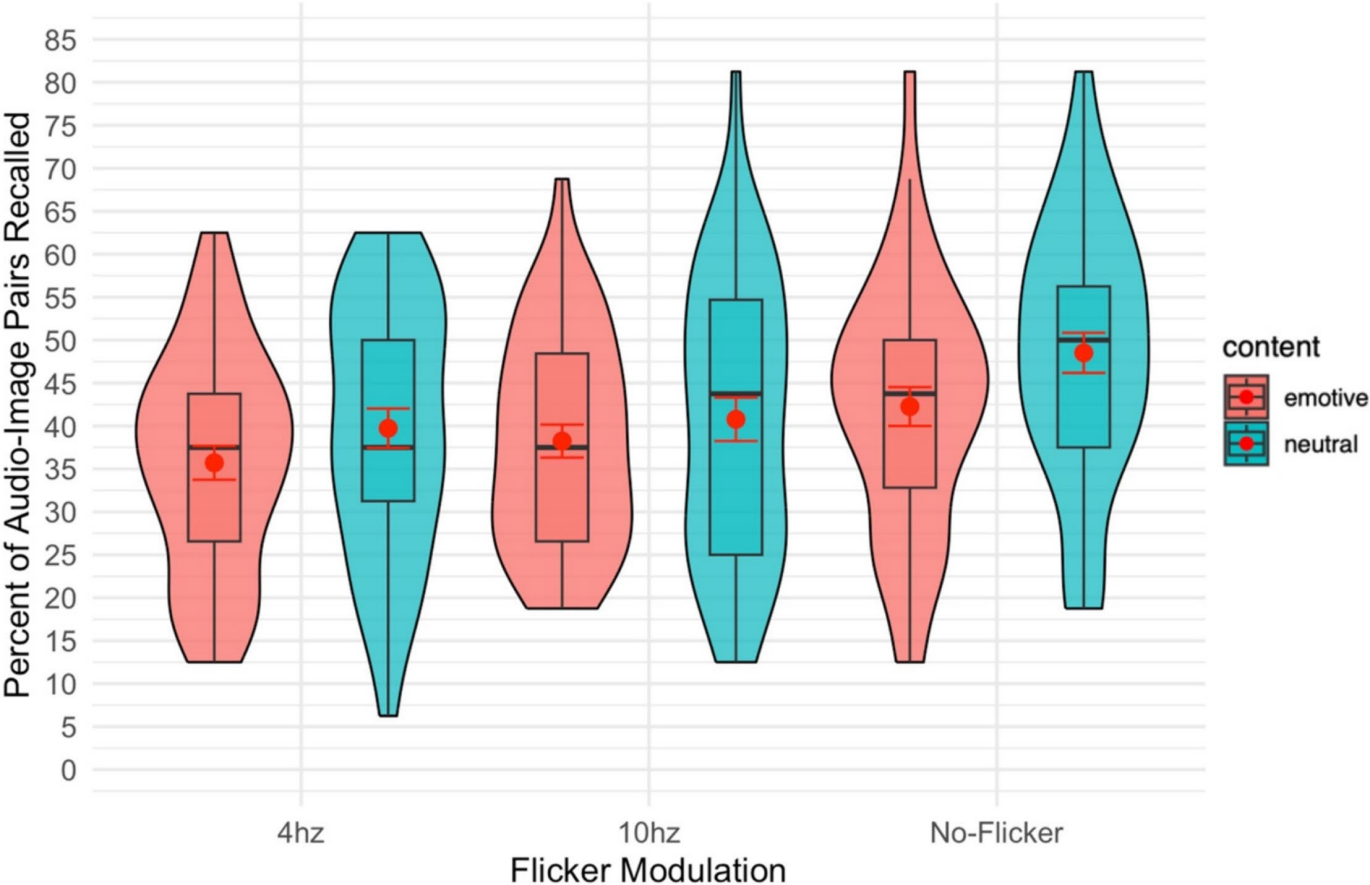
Violin plot of the percent of audio-image pairs recalled overlayed with a box plot. The red dot gives the mean and the red error bars represent the standard error.

There was a significant main effect of modulation frequency on recall accuracy [*F*(2,82) = 9.91, *p* < 0.001, $\eta_{p}^{2}$ = 0.195]. Pairwise comparisons revealed that performance was significantly better in the no modulation (not flickering) condition than in either the 10 Hz (*p* = 0.002, Cohen’s *d* = 0.52) or 4 Hz (*p* < 0.001, *d* = 0.67) modulation conditions (see Figure 2).

There was a significant effect of emotionality (emotive vs. neutral images) on recall of audio-image pairs. [*F*(1,41) = 29.35, *p* = 0.012, $\eta_{p}^{2}$ = 0.145]. This effect was such that image-audio pairs including *non-emotive* images were recalled better than those including emotive images.

There was no significant interaction between the image/audio frequency moderation and the content (emotive/neutral) of the images [*F*(2,82) = 0.48, *p* = 0.623, $\eta_{p}^{2}$ = 0.011].

To match the analysis of Wang et al. (2018) the analysis was repeated excluding those who performed the task at less than chance level (10 participants). This did not have any meaningful impact on the results. There was still a significant effect of modulation frequency [*F*(2,62) = 7.12, *p* = 0.002, $\eta_{p}^{2}$ = 0.019] and emotionality (emotive vs. neutral images) [*F*(1,31) = 13.16, *p* = 0.001, $\eta_{p}^{2}$ = 0.298], and no significant interaction between the terms [*F*(2,62) = 0.20, *p* = 0.821, $\eta_{p}^{2}$ = 0.006]. The direction of these results was the same as above.

### 4.2 Confidence

In line with the accuracy data, there was a significant main effect of modulation frequency [*F*(2,82) = 10.62, *p* < 0.001, $\eta_{p}^{2}$ = 0.206] on rated confidence of recall of the audio-image pair. Pairwise comparisons revealed that performance was significantly better in the no modulation (not flickering) condition than in either the 10 Hz (*p* = <0.001) or 4 Hz (*p* < 0.001) modulation conditions. There was no significant main effect of emotive content [*F*(1,41) = 0.28, *p* = 0.600, $\eta_{p}^{2}$ = 0.007].

### 4.3 Square

**Figure 3:**
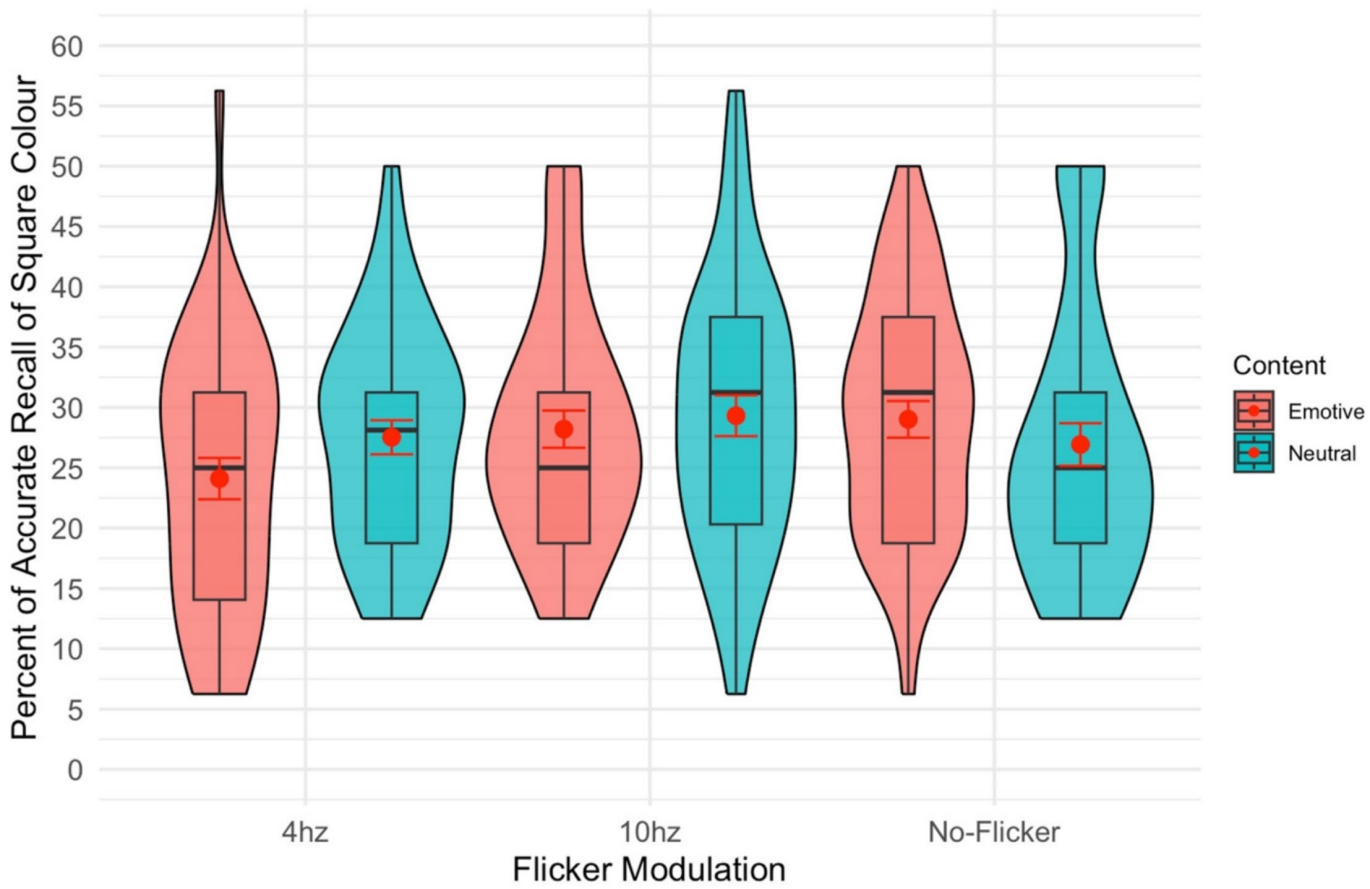
Violin plot of the percent of the time the colour of the square surrounding the stimuli was recalled. The red dot gives the mean and the red error bars represent the standard error.

There was no significant main effect of modulation [*F*(2,82) = 2.74, *p* = 0.070, $\eta_{p}^{2}$ = 0.063; see Figure 3] on participants’ ability to recall the colour of the square surrounding the image. The insignificant trend follows the above recall results, such that performance was generally better in the no-modulation condition. There was also no significant main effect of content [*F*(1,41) = 0.17, *p* = 0.681, $\eta_{p}^{2}$ = 0.004].

## 5 Discussion

The current study failed to replicate previous findings demonstrating improved recall for stimuli synchronously modulated at theta frequency in a multi-sensory associative learning task (Clouter et al., 2017; Wang et al., 2018). We are therefore unable to add to the weight of evidence that theta phase synchronisation plays a causal role in associative binding memory formation. There are two primary possible explanations for the discrepancy between current and previous findings; firstly that we successfully modulated neural oscillatory power and that the theta induced memory effects is limited to “clean” lab environments and neutral stimuli. Secondly, methodological limitations of the current study mean we may have failed to entrain target brain regions. We will consider these possibilities, lessons learned from the study and opportunities for future research, in turn.

The current experiment was performed entirely online, due to restrictions enforced during the COVID-19 pandemic. Although this offers an excellent test of the ecological validity of theta-modulating stimuli for memory gains, it also provides several possible explanations for this failure to replicate. In the various phase offsets in theta-modulated stimuli investigated by Clouter et al. (2017) the differences between the phase of the auditory and visual stimuli were as small at 62.5 ms between the 0° and 270°/90° offsets. Yet the impact these small changes in timing had on the recall effect was significantly detrimental. In remotely-delivered experimental paradigms, the error control over presentation asynchronies is much lower than in-lab, where the presentation equipment, processing speed, cable length etc. are fixed. Error in timing in Pavlovia or on different devices, may have led to unintentional offsets in stimulus timing, preventing any memory enhancement or indeed creating the deficit in modulated stimuli observed here. This lack of milli-second level precision is major limitation of the current study which may explain the failure to replicate the theta induced memory effect (Clouter et al., 2017; Wang et al., 2018). Additionally, as participants completed the tasks from home, there was likely a lot of variation in ambient light and how large a percentage of the participants’ visual field the stimuli occupied which may reduce visual theta entrainment. In addition, the usual issues of online studies of distractions and adherence to task instructions apply here. To mitigate these risks, we spoke to participants over the phone before participating to emphasise the importance of maintaining a distraction-free environment and following task instructions diligently. However, the aversive content of some images may have discouraged participants from focusing on the images being displayed or engaging in the task. Future online studies could aim to motivate task adherence by financially rewarding the “highest” performing participants. Participants were asked to take part at specified times of the day and the length of time taken to take part was monitored. Without any direct EEG recording, we are unable to confirm whether we successfully entrained neuronal populations in our targeted sensory regions to a synchronised theta frequency. However, external sensory entrainment is thought to be a universal and robust feature of sensory neurons, so to the extent that stimulus timing was maintained via remote presentation and attended to, one would hope that this was achieved.

We compared three modulation conditions; 4 Hz (theta) synchronised flickering of visual luminance and auditory volume, 10 Hz (alpha) synchronised flickering of visual luminance and auditory volume and a non-flicker condition. One explanation of the current results is that participants simply found the flickering stimuli distracting and discordant. There is research showing that even low frequency flickering stimuli can lead to visual discomfort (Patterson Gentile and Aguirre, 2020). Indeed, during piloting and via qualitative feedback, participants reported finding the task fairly aversive, due both to the negatively valence images and the discordant effects of stimulus modulation. It is possible that any gains from theta modulation may be nullified by distraction and participant distress, particularly with aversive stimuli as employed here are used.

Importantly, we should consider the interaction between *endogenous* rhythms and external perturbation. A study attempting to entrain endogenous gamma-band oscillations to visual flicker found that using flickering visual stimuli did not influence the dynamics of ongoing gamma oscillations (Duecker et al., 2021). Rather, the visual flicker induced a distinct neuronal response that co-exists alongside the endogenous gamma oscillations in a different visual area. Additionally, studies investigating alpha-band oscillations have also highlighted that stimulus locked (entrained) and intrinsic alpha rhythms may be distinct processes (Gundlach et al., 2020; Keitel et al., 2019). Alongside this, there is inter-individual variation in both periodic and aperiodic components the EEG signal and the peak of the periodic alpha and theta components may vary between participants. That is, 4 Hz stimulation may be variably effective at inducing greater theta power in different individuals. Whether using visual/auditory flickers in theta frequency indeed entrains ongoing endogenous brain oscillations, impacts the interpretation of studies using entrainment to demonstrate a causal role of endogenous theta oscillations (Clouter et al., 2017; Wang et al., 2018). Disentangling these effects will require further investigation directly measuring EEG signals. For example. Using EEG to profile (a)periodic signal components and individualise stimulus modulation may yield superior effects to those observed here.

In the current study we investigated how the content of the memory impacted later recall. We found that irrespective of the flicker condition, audio-image pairs including negatively-valanced images were recalled at a lower rate. While it is generally agreed that emotionally arousing events are more frequently recalled (Buchanan and Adolphs, 2002; Cahill and McGaugh, 1995; Hamann, 2001), memory for items and contexts associated with negative emotion may be weakened (Bisby and Burgess, 2014; Bisby and Burgess, 2017; Brewin et al., 2010). Findings from Cahill and McGaugh (1995) and Cahill and McGaugh (1998) found emotionally arousing experiences preferentially enhance memory for central details, while peripheral or associative elements may not benefit as strongly from this effect. A recent review of the complex manner in which negative emotion impacts memory performance (Bisby and Burgess, 2017) summarised negative item recall is enhanced due to amygdala up regulation (Phelps and LeDoux, 2005), whilst memory for associated items is disrupted due to reduced hippocampal activity (Bisby et al., 2016). The current study therefore adds to the evidence showing that negative emotive content attenuates recall of associated items. As there was no interaction with the modulation conditions in the current study and no neural data, we cannot add to the literature detangling the mechanisms of this effect. However, future studies could use neural entrainment techniques to investigate if disruption in hippocampal neural oscillations are responsible for the impairment in the recall of items associated with negative content.

A study looking at immediate emotional memory enhancement (Talmi and McGarry, 2012) found the advantage for emotional stimuli was abolished when attention to emotive and neutral stimuli was matched. The multi-sensory associative learning task is very difficult to perform well (average performance was 40.87%; chance level = 25%) and so one explanation of these results may be that the participants’ capacity for attention was overwhelmed.

Additionally, emotive events are often semantically related which can also detriment recall of recently experienced emotive stimuli (Talmi and McGarry, 2012). The inclusion of emotive stimuli is a key methodological difference between the current study and previous studies which found a theta induced memory effect (Clouter et al., 2017; Wang et al., 2018). Emotive memories are known to involve some different neural networks to neutral stimuli (Hamann, 2001). In future studies, we would recommend investigating neutral and emotive stimuli in separate blocks, rather than randomly interspersed as they are here, to avoid any contamination of the effect of the emotive images. A final methodological difference that should be noted is the use of movies in prior studies which found a theta induced memory effect (Clouter et al., 2017; Wang et al., 2018), whilst here still images were used. The decision to use still images was made to allow the use of a thoroughly validated set of emotive stimuli (IAPS). However, it should be noted that using still images may have made the stimuli less naturalistic.

In conclusion, we were unable to replicate the finding that flickering visually stimuli at theta frequency improves recall. Further research is need addressing the methodological issues raised here. Neural entrainment using visual/auditory stimuli offers an exciting methodology to explore the oscillatory mechanisms that may underlie memory and other cognitive processes. Further, utilisation of this effect could potentially be useful for enhancing recall of episodic memory in those with memory disorders or even enhancing memory retrieval as required for memory reconsolidation (Elsey et al., 2018; Walsh et al., 2018). However, further assessing the robustness of theta modulatory effects on memory to stimulus salience and valence will be paramount to assessing whether the effect could have real-world application.

## Data Availability

The raw data supporting the conclusions of this article will be made available by the authors, without undue reservation.
